# Furosemide drug as a corrosion inhibitor for carbon steel in 1.0 M hydrochloric acid

**DOI:** 10.1038/s41598-024-58713-4

**Published:** 2024-04-20

**Authors:** Samir Abd El Maksoud, Abd El Aziz Fouda, Haby Badawy

**Affiliations:** 1 Department of Chemistry, Faculty of Science, Port Said University, Port Said, Egypt; 2 Department of Chemistry, Faculty of Science, Mansoura University, Mansoura, 35516 Egypt

**Keywords:** Furosemide, Carbon steel, Potentiodynamoc, Impedance, HCl, Corrosion, Electrochemistry

## Abstract

Furosemide (4-chloro-2-furan-2-ylmethylamino-5-sulfamoylbenzoic acid) was examined as an inhibitor for the corrosion of carbon steel (CS) in 1.0 M HCl. The investigation included mass loss (ML) and electrochemical techniques: potentiodynamic polarization (PP), electrochemical impedance spectroscopy (EIS), and electrochemical frequency modulation (EFM). The efficiency of inhibition rises with increasing Furosemide concentration and temperature. This compound follows the Temkin isotherm with good fit. The presence of varying quantities influences both anodic metal dissolution and cathodic hydrogen evolution. Scanning electron microscopy (SEM), atomic force microscopy (AFM), X-ray photoelectron spectroscopy (XPS), and Fourier transform infrared spectroscopy (FT-IR) were used to detect the effect of the compound on the CS surface. The molecular inhibitory effect of Furosemide was demonstrated using quantum chemical calculations, and the molecular simulation results demonstrated the adsorption on the carbon steel surface.

## Introduction

The study of carbon steel corrosion in acidic destructive solutions is essential in industry^1^. The acidic solution is widely used to: (1) remove scale in industrial operations; (2) treat water plants; and (3) pickle oil recovery and petrochemical processes. Acid aqueous solutions are among the most corrosive media, and CS is extensively used in pipelines, in the oil and gas industries. Inhibitors are used to prevent CS corrosion^2^. The corrosion inhibition efficacy of the compounds is related to the adsorption capabilities, which form an adsorbing layer on CS (physisorption or chemisorption)^3^. The adsorption is influenced by the nature and state of the metal surface, the type of corrosive media, and the chemical structure of the inhibitor^4,5^.

Organic compounds contain heteroatoms such as sulphur, nitrogen, oxygen, and an aromatic ring, were extensively utilized as corrosion inhibitors^6^. Most organic compounds are toxic and harmful to the environment^7–13^. Because of the toxicity of these organic inhibitors, there is an opportunity to investigate the application of environmentally non-toxic inhibitors^14–16^. It is critical to use friendly acid corrosion inhibitors that are low-cost, non-hazardous, and efficient^17–25^. The corrosion inhibition activity of several medications has been studied in order to determine their potential use as a competitive class of green corrosion inhibitors. Many medicines' corrosion inhibition performances have been investigated^26–46^. The current research aims to evaluate Furosemide as a corrosion inhibitor for CS corrosion in 1.0 M HCl. The inhibition of CS corrosion in 1.0 M HCl was determined and confirmed using chemical and electrochemical techniques. The adsorbed film on the metal surface was characterized using AFM, SEM, EDX, FT-IR, and XPS analyses.

## Materials and methods

### Materials

Chemical composition of carbon steel: 0.14% C, 0.52% Mn, 0.05% P, 0.02% Si, 0.03% Cr, 0.02% Ni, 0.002% P, 0.04% S, 0.02% Cu, and the rest Fe. The aggressive solution of 1.0 M HCl was prepared by dilution with bidistilled water. The inhibitor structure is illustrated in Table 1. The stock solution of Furosemide (1000 ppm) concentration was prepared by dissolving the calculated mass in bidistilled water. The concentrations used ranged from 50 to 300 ppm.

**Table 1:** The molecular structures, name, chemical formula and molecular weight of Furosemide.

Structure	IUPAC name	Molecular weight	Chemical formula
	4-Chloro-2-(furan-2-ylmethylamino)-5-sulfamoylbenzoic acid	330.74 g/mol	C_12_H_11_ClN_2_O_5_S

### Methods

#### Mass loss

The test specimens of CS with the dimensions (2 × 2 × 0.1 cm) were used. The samples were abraded with emery sheet grit (sizes 400, 600, 800, and 1200) to ensure the softness of the CS surface and then washed with bidistilled water, degreased with acetone, dried and weighed. The weighted samples were immersed in 100 ml of 1.0 M HCl in the absence and presence of different concentrations of the examined compound. The time of immersion was recorded at various temperatures; the temperature was controlled in the water by the thermostatic bath with an accuracy of ± 0.1 °C. After the immersion time, CS sheets were removed from the solution, washed with bidistilled water, dried, and weighted. Experiments were repeated three times with diverse samples to confirm the repeatability of the results.

The IE% and surface coverage (θ) of the inhibitor were calculated by Eq. (1)

1 $$\mathrm{IE\%}=\left(1-\left[\frac{\mathrm{ W}}{{\text{Wo}}} \right] \right)\times 100 =\uptheta \times 100$$

where W_o_, and W values are the mass loss without and with different concentrations of the inhibitor, respectively.

#### Electrochemical techniques

Potentiodynamic polarization, electrochemical impedance spectroscopy, and the electrochemical frequency modulation technique were utilized to detect the corrosion behaviour of CS in 1.0 M HCl. All the experiments were carried out in glass cells with three electrodes. The counter electrode was a Pt electrode, the reference electrode was a saturated calomel electrode, and the working electrode was CS. The working electrode area was 1.0 × 1.0 cm. It was rinsed, polished with emery paper of different grades, washed and degreased with acetone, then washed with bidistilled water. All the experiments were carried out at 25 ± 1 °C. The open-circuit potential was started for 30 min and then recorded until the steady state of the specimens was reached. All the electrochemical experiments were carried out using a potentiostat/galvanostat from Gamry Instrument Series G 750.

##### Potentiodynamic technique (PP)

In this method, the potential was applied from − 0.750 to − 0.250 V at a scan rate of 1 mV s^−1^, and the corrosion current density (i_corr_) was estimated. The IE% and θ can be calculated from Eq. (2)

2 $$\mathrm{IE\%}=\left(1-[\frac{ {{\text{i}}}_{{\text{corr}}\left({\text{inh}}.\right)}}{{{\text{i}}^\circ }_{{\text{corr}}\left({\text{free}}\right)}} ] \right)\times 100 =\uptheta \times 100$$

I^o^_corr_ and i_corr_ are the current densities without and with inhibitors, respectively.

##### Electrochemical impedance spectroscopy (EIS)

Impedance measurements were performed in the frequency range from 100 kHz to 0.1 Hz using 5 mV amplitude. IE% and θ were estimated from Eq. (3)

3 $$\mathrm{IE\% }=\left(\frac{{{\text{R}}^\circ }_{{\text{p}}}- {{\text{R}}}_{{\text{p}}}}{{{\text{R}}^\circ }_{{\text{p}}}}\right)\times 100 =\uptheta \times 100$$

R^∘^_p_ and R_p_ are the polarization resistances with and without inhibitors, respectively. The double-layer capacitance (C_dl_) values in the presence of different concentrations were determined from Eq. (4):

4 $${{\text{C}}}_{{\text{dl}}}=\frac{1}{2\uppi {{\text{f}}}_{{\text{max}}}{{\text{R}}}_{{\text{ct}}}}$$

where f_max_ is the value of maximum frequency.

##### Electrochemical frequency modulation technique (EFM)

EFM can be used as a fast and non-destructive method for CS corrosion without knowledge of the Tafel slope. EFM technique with amplitudes of 10 mV and two sine waves of 2 and 5 Hz High peaks are used to calculate the current density (i_corr_), Tafel slope (βc and βa) and the causality factors (CF-2 and CF-3).

#### Surface investigations

##### Scanning electron microscopy

After dipping the CS specimen in 1 M HCl without and with 300 ppm Furosemide for a day at 25 °C, the surface of the CS specimen was investigated. A scanning electron microscope (SEM; JEOL JSM-5500, Japan) was used. SEM images of the metal surface were taken at a magnification of 2000.

##### Atomic force microscopy (AFM)

The outer surface roughness can be assessed using an AFM instrument that creates a topographic surface map with a distinct resolution. Surface roughness results from variations in a surface's ideal shape induced by corrosion or inhibitor adsorption. Thermo Fisher Nicolet IS10 (scanning probe microscope) was employed.

##### X-ray photoelectron spectroscopy (XPS)

These studies were carried out using a highly efficient system that uses XPS to identify the binding energies of various bonds discovered on the carbon steel surface, allowing the adsorbed atoms and functional groups on the metal surface to be determined. K-ALPHA (Thermo Fisher Scientific, USA) was used.

##### Fourier-transform infrared spectroscopy (FTIR)

FTIR spectra of pure solutions of the inhibitor and carbon steel sheets immersed in 1 M HCl plus the optimal concentration of the inhibitor for 24 h were measured using a PerkinElmer 1600 spectrophotometer.

#### Simulation analysis

The quantum chemical parameters were found by MSD Mol440^47^, which use efficient density theory (DFT). The chemical quantum parameters, the highest occupied molecular orbital (E_HOMO_) and the lowest unoccupied molecular orbital (E_LUMO_), dipole moments (µ), energy gaping (ΔE), hardness (η), softness (σ), ionization potentials (I), and electronegativity (X) were calculated for the inhibitor.

## Results and discussion

### Mass loss (ML) method

#### Effect of inhibitor concentrations

**Figure 1:**
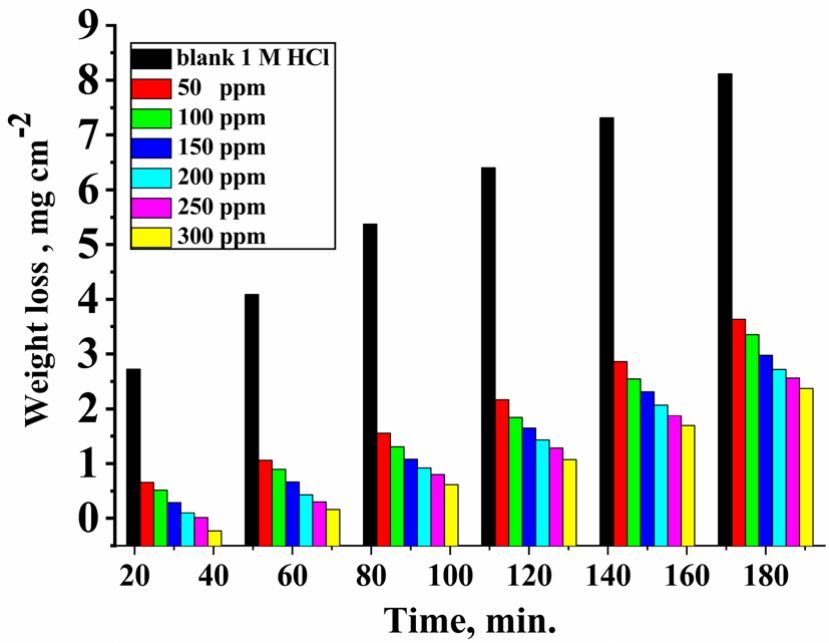
Weight loss vs. time for the corrosion of CS in 1.0 M HCl without and with various concentrations from Furosemide at 298 K.

Figure 1 represents the relation between mass loss and time for CS in the absence and presence of various concentrations of Furosemide in 1.0 M HCl at 25 °C. The data derived from the mass loss method are presented in Table 2. The data revealed that the mass loss and the corrosion rate decreased with increasing concentrations. The increase in the IE% was due to the barrier formation of inhibitors on the metal surface, which decreases the dissolution of the metal^48^. IE% for CS corrosion was evaluated from Eq. (1).

**Table 2:** Mass loss data for CS corrosion at 120 min in 1.0 M HCl without and with various concentrations from Furosemide at 298 K.

Conc. (ppm)	Temp. (K)	ML (mg cm^−2^)	k_corr_ ×10^3^ (mg cm^−2^ min^−1^)	θ	IE %
Blank	298	6.4	53.33	–	–
50		1.87	15.64	0.706	70.6
100		1.65	13.82	0.740	74.0
150		1.46	12.21	0.771	77.1
200		1.32	11.02	0.793	79.3
250		1.17	9.78	0.816	81.6
300		1.07	8.93	0.832	83.2

#### Effect of temperature

**Figure 2:**
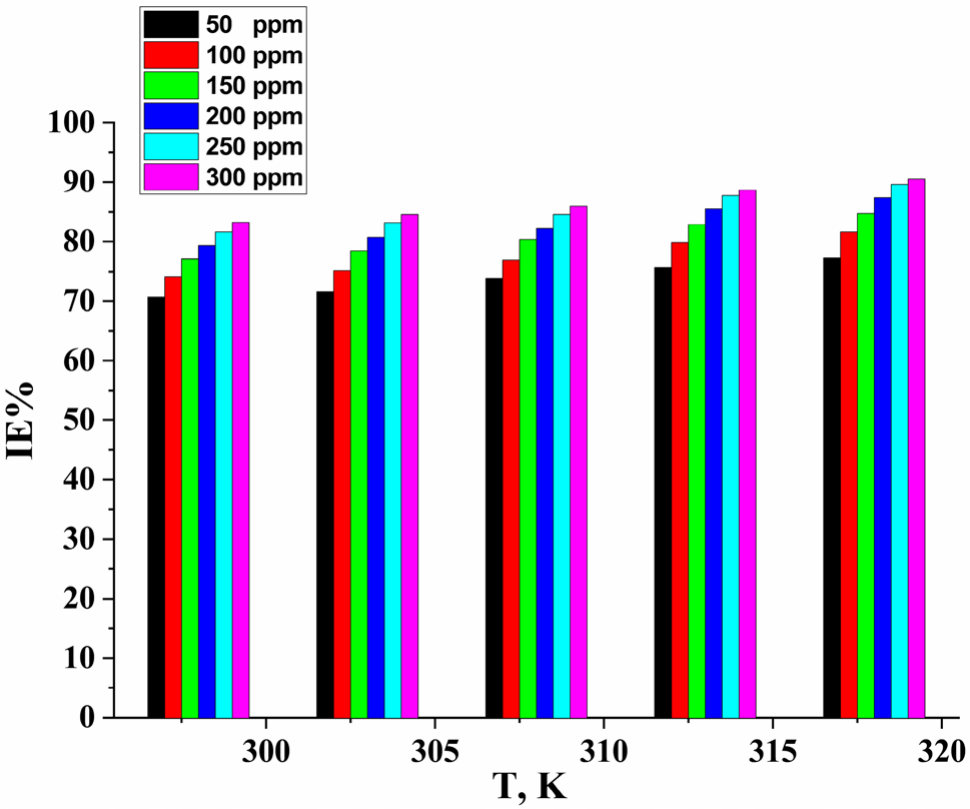
Effect of temperature on the IE% at various concentrations from Furosemide for CS in 1.0 M HCl solution at different temperatures.

The ML method was used to study the effect of temperature on the corrosion rate of CS in 1.0 M HCl containing different concentrations. Figure 2 represents the effect of temperature on the IE% at different concentrations of the studied compound on CS dissolution in a 1.0 M HCl solution. The results obtained are illustrated in Table 3. From the results, we can conclude that the corrosion rate increases in the corrosive medium with increasing temperature. The inhibition efficiency increased with increasing solution temperature, which indicates the chemical absorption type of the inhibitor on the CS surface^49^.

**Table 3:** Mass loss data for CS corrosion at 120 min in 1.0 M HCl without and with various concentrations from Furosemide at various temperatures 303–318 K.

Conc. (ppm)	Temp. (K)	ML (mg cm^−2^)	k_corr_ × 10^3^ (mg cm^−2^ min^−1^)	θ	IE %
Blank	303	7.01	58.42	–	–
50		1.98	16.57	0.716	71.6
100		1.73	14.49	0.751	75.1
150		1.51	12.59	0.784	78.4
200		1.34	11.24	0.807	80.7
250		1.17	9.80	0.832	83.2
300		1.07	8.99	0.846	84.6
Blank B	308	9.62	80.1	–	–
50		2.52	21.0	0.738	73.8
100		2.21	18.49	0.769	76.9
150		1.88	15.72	0.803	80.3
200		1.71	14.25	0.822	82.2
250		1.48	12.34	0.846	84.6
300		1.35	11.25	0.859	85.9
Blank	313	12.82	106.8	–	–
50		3.11	25.96	0.756	75.6
100		2.58	21.55	0.798	79.8
150		2.18	18.24	0.829	82.9
200		1.85	15.46	0.855	85.5
250		1.57	13.10	0.877	87.7
300		1.45	12.08	0.886	88.6
Blank	318	15.42	128.5	–	–
50		3.59	29.95	0.772	77.2
100		2.90	24.18	0.816	81.6
150		2.41	20.09	0.847	84.7
200		1.99	16.63	0.873	87.3
250		1.64	13.68	0.896	89.6
300		1.50	12.50	0.905	90.5

#### Activation thermodynamic parameters

The corrosion rate of the reaction is influenced by the temperature; Eq. (5) specifies this relation according to Arrhenius^50,51^:

5 $$ {\text{k}}_{{{\text{corr}}}}  = {\text{A}}\;{\text{exp}}(E_{a}^{*} /{\text{RT}}) $$

where A is the Arrhenius constant, E^*^_a_ is the activation energy, T is the absolute temperature and R is the gas constant.

**Figure 3:**
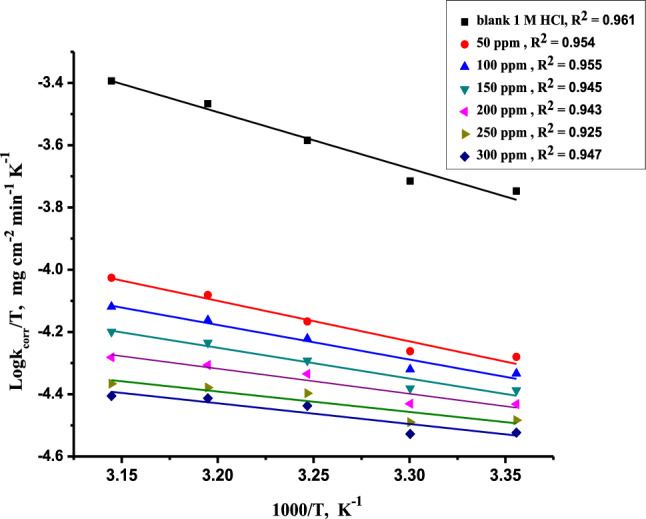
Arrhenius plots for dissolution of CS in 1.0 M HCl solution with and without different concentrations of Furosemide at different temperatures (298–318 K).

Figure 3 shows the plot of log k_corr_ vs. 1/T with straight lines; from the line’s slopes, we can calculate E^*^_a_. The values of E^*^_a_ are calculated and reported in Table 4.

**Table 4:** Activation thermodynamic data for dissolution of (CS) in 1.0 M HCl in the absence and presence of various doses of Furosemide at different temperatures (298–318 K).

Conc. (ppm)	E_a_* (kJ mol^−1^)	∆H* (Kj mol^−1^)	−∆S* (J mol^−1^ K^−1^)
0.0	37.2	34.6	153.6
50	24.5	21.9	201.8
100	19.2	16.7	219.9
150	17.3	14.7	227.4
200	15.4	12.8	234.4
250	13.8	11.2	240.2
300	13.1	10.5	243.1

**Figure 4:**
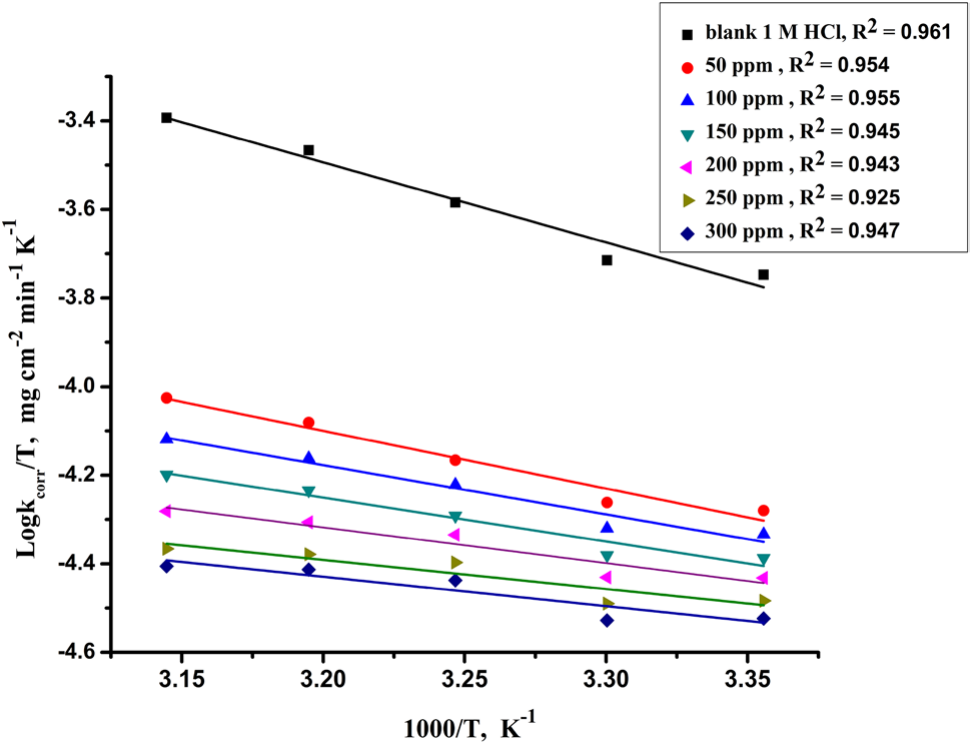
Plotting the transition state of Log (kcorr/T) vs. (1/T) for dissolution of CS in 1.0 M HCl solution in the absence and presence of various doses of Furosemide compound at various temperatures (298–318 K).

From the transition state theory as shown in Eq. (6):

6 $$ {\text{k}}_{{{\text{corr}}}} = {\text{ RT}}/{\text{ Nh exp }}\Delta {\text{S}}^{*} {\text{exp}}^{{ - \, \Delta {\text{H}}*/{\text{RT}}}} $$

where ΔS^*^ and ΔH^*^ are the entropy and enthalpy of activation, respectively. Figure 4 shows the Plot of log kcorr/T vs. 1/T, giving straight lines from their slopes ΔH^*^ can be calculated and reported in Table 4. The results indicate that ${E}_{a}^{*}$ values decrease in the presence of an inhibitor compared to their absence, indicating chemical adsorption. The positive values of ΔH^*^ indicate the endothermic activation process, which confirms the chemical adsorption of the investigated inhibitor on the CS surface^52^.

#### Adsorption isotherms

The type and charge of the metal, the structure of the inhibitor, and the composition of the electrolyte all influence inhibitor adsorption on the metal surface^53^. The Temkin isotherm, as illustrated in Eq. (7), well matches the data:

7 $$ {\text{q}} = \left( {{2}.{3}0{3}/{\text{a}}} \right){\text{Log K}}_{{{\text{ads}}}} + \left( {{2}.{3}0{3}/{\text{a}}} \right){\text{Log C}} $$

**Figure 5:**
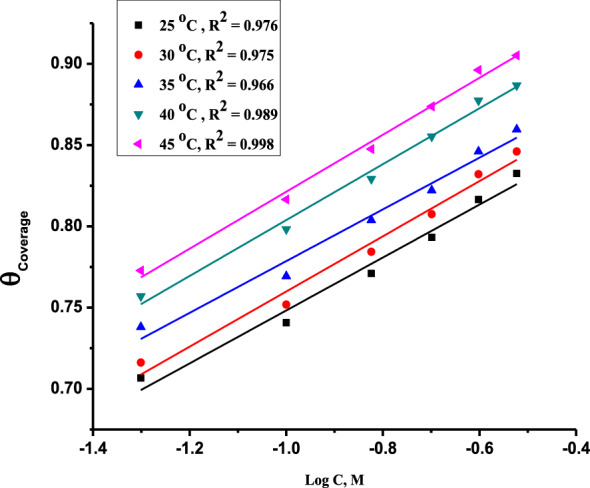
Plots of θ vs. Log C for the Furosemide compound on CS surface in 1.0 M HCl at various temperatures (298–318 K).

"a" is the CS's heterogeneous factor (a particle interaction factor), and C is the inhibitor concentration. Plotting θ vs. log C for inhibitors at different temperatures in Fig. 5 results in straight lines with log Kads slopes and intercepts (2.303/a).

The free energy of adsorption (G^o^_ads_) was calculated using Kads in Eq. (8) as follows:

The Temkin adsorption isotherm was very good, with a correlation factor R^2^ close to 0.978–0.996. The values of "a", K_ads_ and ΔG^o^_ads_ were listed in Table 5. K_ads_ in Eq. (8) was used to determine the free energy of adsorption (ΔG^o^_ads_) as follows:

8 $$ \Delta {\text{G}}^{{\text{o}}}_{{{\text{ads}}}} = - {\text{RT ln }}\left( {{55}.{\text{5 K}}_{{{\text{ads}}}} } \right) $$

where 55.5 is the molar dosage of H_2_O in the bulk of the solution (mol L^−1^). The calculated data showed that these studied compounds were adsorbed on CS through the lone pair of electrons of heteroatoms (S, N, or O atoms). The ΔG^o^_ads_ and K_ads_ values increase with temperature in the range of 298–318 K, also indicating the chemical adsorption of this inhibitor on the CS surface.

**Table 5:** Adsorption parameters of Temkin isotherm for the (CS) surface in 1.0 M HCl for Furosemide compound at (298–318 K).

Temp. (K)	Log (K_ads_ M^−1^)	A	− ∆G^°^_ads_ (kJ mol^−1^)	∆H^◦^_ads_ (kJ mol^−1^)	− ∆S^◦^_ads_ (J mol K^−1^)
298	5.594	14.0	41.8	77.6	397.9
303	5.708	14.2	43.2		398.8
308	5.909	14.5	45.1		398.4
313	6.168	14.7	47.4		399.4
318	6.439	15.0	49.8		400.7

**Figure 6:**
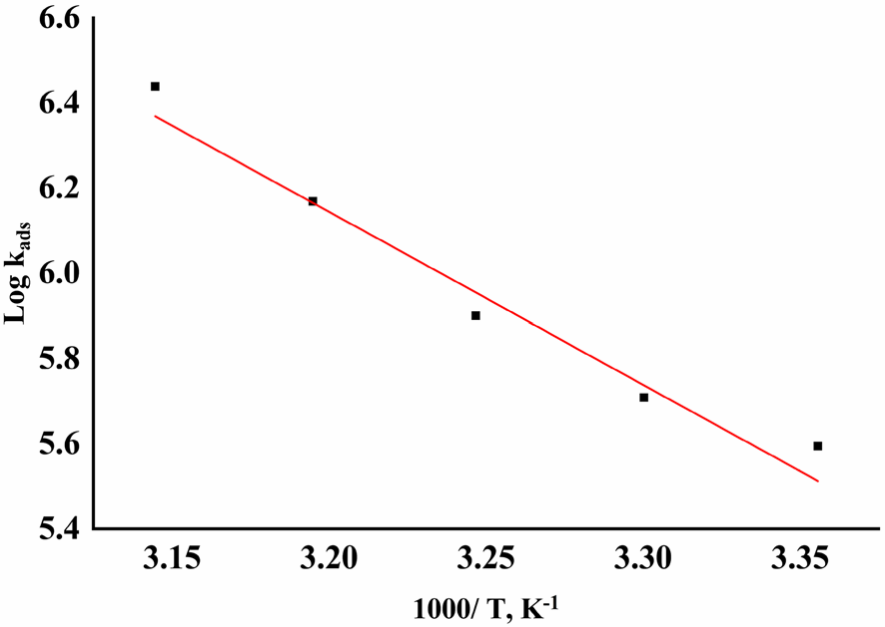
Plots of variation of Log K_ads_ vs. 1/T for the adsorption of various doses of Furosemide compound on the CS surface in 1.0 M HCl at (298–318 K).

Figure 6 as well as Eq. (9), can detect the heat adsorption (∆H^o^_ads_)^54^.

9 $$ {\text{Log}}\;{\text{K}}_{{{\text{ads}}}} = - \Delta {\text{H}}_{{{\text{ads}}}}^{ \circ } /2.303{\text{RT}} $$

where (− ∆H^o^_ads_/2.303R) is the slope of the straight-line Log K _ads_ vs. 1/T, ∆S^o^_ads_ obtained by Eq. (10):

10 $${\Delta {\text{G}}}_{{\text{ads}}}^{{\text{o}}}={\Delta {\text{H}}}_{{\text{ads}}}^{{\text{o}}}-\mathrm{ T}{\Delta {\text{S}}}_{\mathrm{ads }}^{{\text{o}}}$$

Data in Table 5 indicated that ΔG°_ads_ values were greater and around − 40 kJ mol^−1^ dependent on adsorbing by inhibitors on CS surface chemisorption, the coordination of electrons of active groups of the inhibitor molecules to unfilled d-orbitals of CS^55^. The negative values of the free energy of adsorption (ΔG°_ads)_ express the spontaneity of the adsorption. The values of ΔH°_ads_ in the present study are higher than 40 kJ mol^−1^, which means the chemisorption process^56^. The unshared electron pairs (S, N, and O atoms) might depend on the metal surface of CS. The "a" +ve values are due to the interaction of the adsorbed layers on the CS surface. Furthermore, the values of K_ads_ reveal that the increase in the adsorption equilibrium caused by an increase in temperature (Table 5). Entropy values were negative, revealing that the adsorption of the inhibitor was associated with adsorption rather than desorption on the metal surface. Table 5 shows the increase in ∆S^o^_ads_ values as the disorders increase owing to the adsorption of H_2_O molecules by the adsorption of inhibitors on the surface of CS^57^.

### Electrochemical techniques

#### Open circuit potential (E_OCP_)

**Figure 7:**
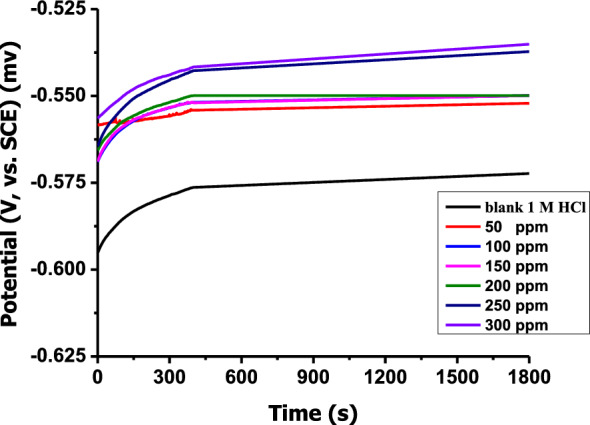
Potential-time curves for CS immersed in 1.0 N HCl solution at 298 K without and with different concentrations from Furosemide.

Figure 7 depicts the change in E_OCP_ over time obtained for C-steel in 1 M HCl in the absence and presence of various doses of Furosemide at 298 k. The figure demonstrates that E_OCP_ in the blank started at − 573 mV and then shifted anodically until the steady state was achieved. In the presence of Furosemide, E_OCP_ began with a significantly positive potential compared to its absence and later shifted anodically. The shift in E_OCP_ becomes more positive as the concentration increases. The highest shift in the value of OCP was 35 mV in the presence of the inhibitor. This behaviour indicates that the corrosion inhibitor functions as a mixed type corrosion inhibitor for C-steel in aggressive 1 M HCl^58,59^.

#### Potentiodynamic polarization (PP)

**Figure 8:**
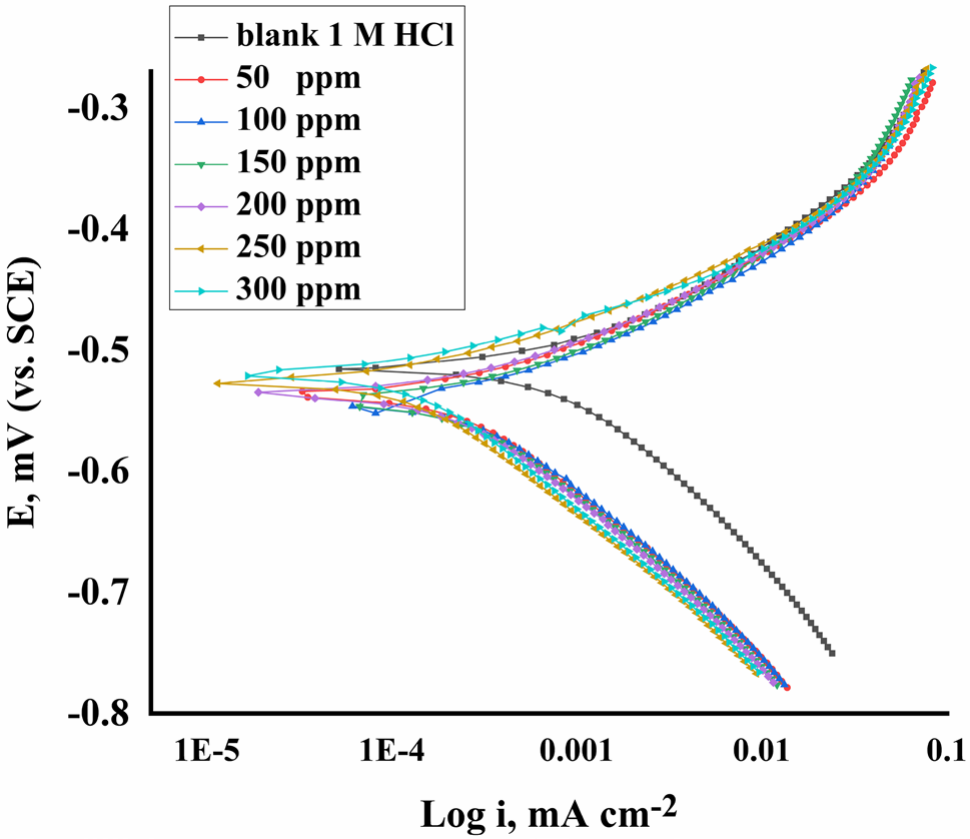
Plotting of PP for dissolution of (CS) in 1.0 M HCl using different doses of Furosemide at 298 K.

Figure 8 shows the polarization performance of the CS in 1.0 M HCl with and without different doses of the inhibitor. The measured data: corrosion current density (i_corr_), corrosion potential (E_corr_), cathodic Tafel slope (β_c_), Tafel slope (β_a_), (θ), and ($\mathrm{IE\%}$) were estimated and reported in Table 6. Moreover, by adding an inhibitor, the corrosion current decreased, indicating that this inhibitor agreed with the suitable inhibitor's behaviour. The slight change in both Tafel slopes and E_corr_ indicates that this investigated inhibitor behaves as a mixed type inhibitor^60^. The Tafel lines were parallel with and without inhibitor, indicating that there is no change in the mechanism.

**Table 6:** Data of PP of CS in 1.0 M HCl without and with various doses of Furosemide at 298 K.

Conc. (ppm)	− E_corr_ (mV vs. SCE)	i_corr_ (μA cm^−2^)	− β_c_ (mV dec^−1^)	β_a_ (mV dec^−1^)	k_corr_ (mpy)	θ	IE %
0	533	1480	210	134	755	–	–
50	522	529	193	108	269.5	0.642	64.2
100	518	480	175	104	245	0.675	67.5
150	520	468	179	106	239	0.683	68.3
200	515	326	190	113	168	0.779	77.9
250	521	212	166	81	111	0.856	85.6
300	520	183	188	76	96	0.876	87.6

#### Electrochemical impedance spectroscopy

**Figure 9:**
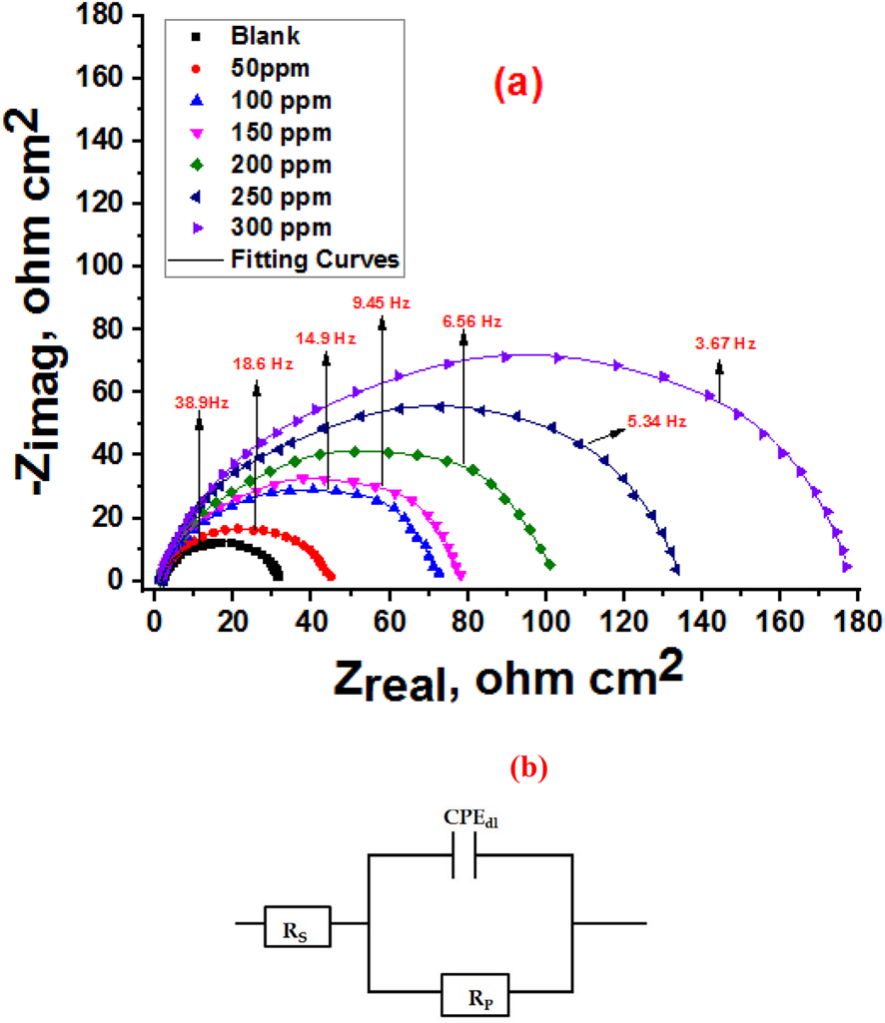
(**a**) EIS spectra of Nyquist for dissolution of CS in 1.0 M HCl without and with various doses of the Furosemide compound at 298 K. (**b**) Electrochemical equivalent circuit used by fitting the EIS data.

Electrochemical impedance spectroscopy (EIS) is a technique used to gain insight into the characteristics of electrochemical processes that occur on the steel surface in acid solution. Figure 9a shows Nyquist plots of carbon steel with and without various amounts of Furosemide in 1.0 M HCl. They are semicircles moving along the real impedance of the x-axis. However, the smoothness of these semicircles deviated slightly from the classic EIS idea. Metal surface heterogeneity and frequency dispersion can also create imperfect capacitance loops^61^. The semicircle radii are changed by the inhibitor concentrations. The radius of the semicircular lines increases in direct proportion to the concentration. The widths of the capacitance loops are greater in the presence of Furosemide than in its absence, demonstrating that inhibitor molecules can greatly promote steel surface anticorrosion^62^. The concentration of inhibitor molecules adsorbing on carbon steel's surface is proportional to its concentration. The double-layer capacitance (C_dl_) from Eq. (11) can be reproduced using CPE^63^:

11 $$\mathrm{C }_\text{dl}= {Y}_{o}({\omega }_{max})\mathrm{ n}-1$$

where ${\omega }_{max}$ represents the frequency corresponding to the maximum value of the imaginary component of the Nyquist plot.

Figure 9b shows the correlated equivalent circuit used to model the CS/HCl interface, where Rs represents the uncompensated solution resistance, the Rp, includes charge transfer resistance (Rct), diffuse layer resistance (Rd), the accumulated species at the CS/HCl interface (Ra), and the resistance of the inhibitor film at the surface^64–67^.

Table 7 shows that R_p_ values increase as inhibitor concentration increases. The R_p_ value varies between 44.1 and 176.9 Ω/cm^2^. This could imply that the inhibitor develops a protective coating on the electrode surface at all concentrations. This could imply that the inhibitor develops a protective coating on the electrode surface at all concentrations^68^. As a result, corrosive ions are successfully kept from coming into contact with the working electrode. At the same time, the addition of inhibitors lowers C_dl_ values by forming a protective layer on the steel surface. The inhibitor exhibits the highest inhibitory efficiency at a concentration of 300 ppm, according to the EIS method. The varying n values in the presence of inhibitors indicate that the heterogeneous properties of the steel surface have changed. This could be related to the adsorption of inhibitor molecules on the electrode surface^69^.

**Table 7:** EIS data for the corrosion of (CS) in 1.0 M HCl without and with various doses of Furosemide at 298 K.

Conc., (ppm)	R_S_ Ω cm^2^)	Y_ο_ (µΩ^−1^ s^*n*^ cm^−2^)	n	R_p_ (Ω cm^2^)	C_dl_ (µF cm^−2^)	θ	IE %	Goodness of fit (χ^2^)
0	1.9	325.6	0.970	30.9	284.4	–	–	8.24 × 10^–3^
50	1.82	221.7	0.852	44.1	82.9	0.314	29.9	9.57 × 10^–3^
100	1.64	201	0.811	75.6	75.5	0.605	60.5	7.44 × 10^–3^
150	1.39	197	0.808	79.4	73	0.628	61.1	6.54 × 10^–3^
200	1.5	168.5	0.792	102.4	65.4	0.712	69.8	9.44 × 10^–3^
250	1.88	174	0.782	135.3	60.7	0.781	77.2	8.78 × 10^–3^
300	1.17	161	0.752	176.2	49.6	0.834	82.5	6.87 × 10^–3^

#### Electrochemical frequency modulation

**Figure 10:**
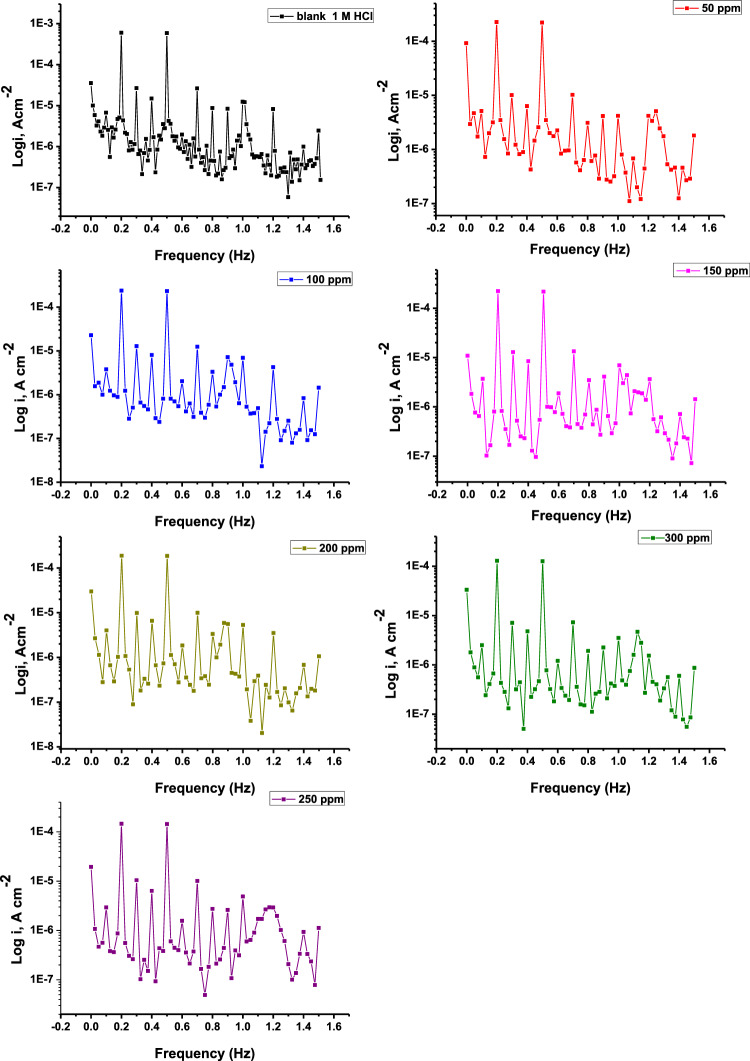
EFM spectra for corrosion of the CSl surface in 1.0 M HCl with and without various concentrations of Furosemide compound at 298 K.

Two sine waves with frequencies of 2 and 5 Hz are supplied to the cell for EFM measurements in the absence and presence of varying doses of the examined substance. The Tafel constants are not required because the results are generated instantaneously with this approach^70^. The output current fluctuates with frequency and is nonlinear. The causality factors (CF-2 and CF-3) acquired from EFM testing are critical because they validate the EFM measurements if their values are near the theoretical values (2 and 3). Harmonic peaks in the output spectrum of the current compromise the data for CR. The larger the current density (i_corr_), the higher the peaks. The EFM spectra of CS in 1 M HCl in the absence and presence of various concentrations are shown in Fig. 10 and the results are displayed in Table 8. As the inhibitor concentration rose, the i_corr_ decreased. Theoretical values are equivalent to testing-derived causality factors^71^.

**Table 8:** Electrochemical parameters from EFM of the CS in 1.0 M HCl without and with various doses of Furosemide at 298 K.

Compound	Conc. (ppm)	i_corr_ (µA cm^−2^)	− β_c_ (mv dec^−1^)	β_a_ (mV dec^−1^)	CF-2	CF-3	k_corr_ (mpy)	θ	IE %
Blank	0	929	191	135	1.8	3.5	480	–	–
Inhibitor	50	400	102	80	1.7	3.2	215	0.569	56.9
	100	380	100	83	2.4	3.1	200	0.591	59.1
	150	280.2	103	84	1.8	3.0	148.1	0.698	69.8
	200	262.1	97	78	1.6	2.8	148.1	0.717	71.7
	250	239.5	97	80	1.8	3	124.7	0.742	74.2
	300	210.6	87	72	1.7	3.1	110.3	0.773	77.3

### Surface investigations

#### SEM examination

**Figure 11:**
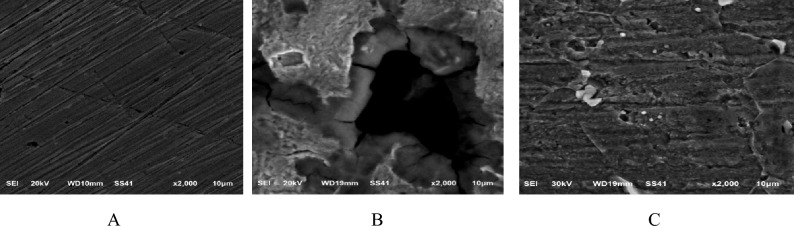
SEM micrographs of (CS) samples (**A**) pure CS sample, (**B**) CS in 1.0 M HCl solution after the immersion for 24 h at 298 K (**C**) CS immersed in 1.0 M HCl solution with 300 ppm of Furosemide for 24 h at 298 K.

C-steel bars were immersed in a corrosive medium containing 300 ppm Furosemide at 298 K for 24 h to study surface morphology^72^. The effect of Furosemide addition on the surface of C steel is depicted in Fig. 11. Figure 11b,c depict the surface of C-steel in corrosive conditions without and with the addition of Furosemide, respectively, demonstrating the production of rust on the metal surface as a result of exposure to corrosive medium oxygen, water molecules, and chloride ions, as described in Eqs. (12) and (13):

12 $${Fe}^{+2}+2{Cl}^{-}\to {FeCl}_{2}$$

13 $${H}_{2}O+{O}_{2}+{FeCl}_{2}\to {2H}^{+}+2{Cl}^{-}+{FeO}_{3}$$

The figure shows the construction of a shielding adsorbed layer on the surface of the C-steel.

#### AFM examination

**Figure 12:**
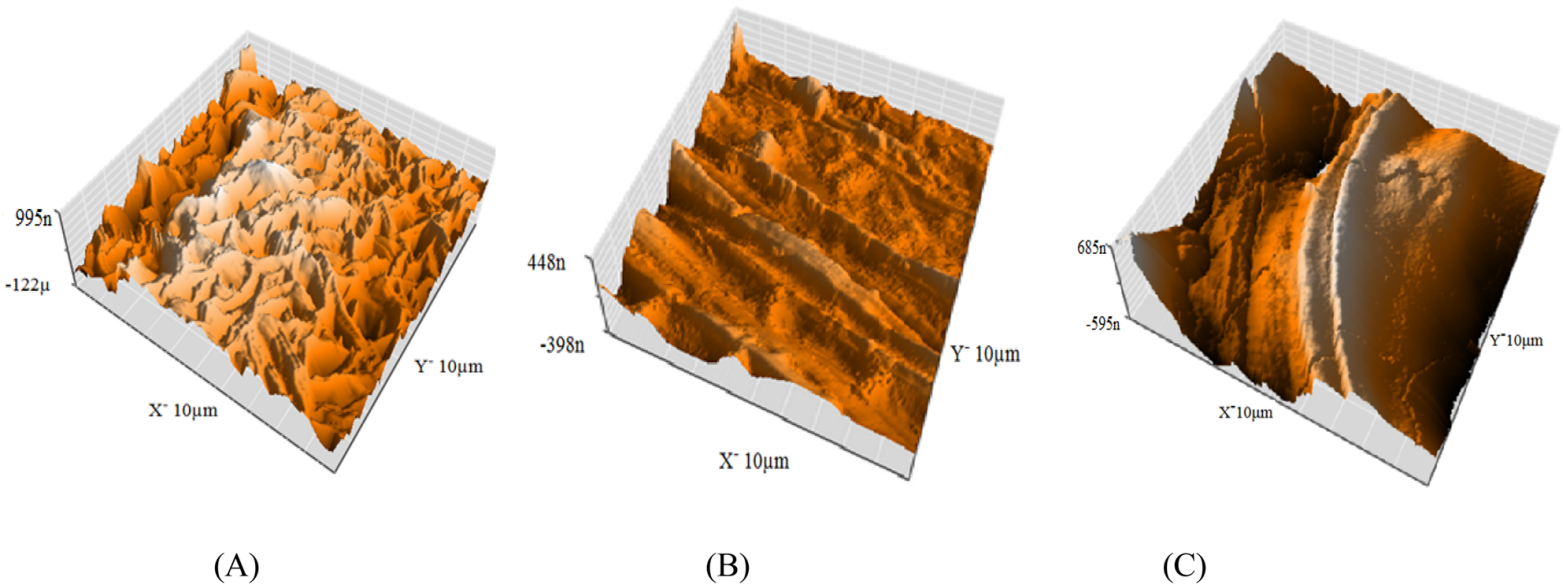
Three-dimensional (3D) AFM images of the CS samples, (**A**) before involvement in acid, (**B**) the CS sample immersed in 1.0 M HCl for 24 h at 298 K (**C**) the CS sample immersion in 1.0 M HCl with 300 ppm of the Furosemide for 24 h at 298 K.

AFM was employed to measure surface image evidence at the nano-to-micro-scale^73^. The morphologies of CS dissolution in a 1.0 M HCl corrosive solution in the absence and presence of a 300 ppm inhibitor are shown in Fig. 12. The root mean square roughness (Rq) determines the average lines, and the average roughness (Ra) explains the mean deviations of all roughness images. Significant corrosion and increased roughness are visible in an AFM image of the CS specimen in 1.0 M HCl. The measured IE% calculated from ML and electrochemical performances were validated and matched with roughness data. The data in Table 9 reveal that in the presence of Furosemide, the metal surface is smoothed due to the adsorption of the compound through the active center sites.

**Table 9:** AFM roughness measurements of the (CS) samples without and with 300 ppm of from the investigated compound after immersion in 1.0 M HCl for 24 h at 298 K.

Specimen	Average roughness (Ra) nm	RMS roughness (Rq) nm
(CS) metal surface (pure)	48.7	63.0
(CS) metal surface + 1.0 M HCl (blank)	271.7	335.6
(CS) metal surface + Furosemide compound	108.2	141.8

#### Examination of X-ray photoelectron spectroscopy (XPS)

**Figure 13:**
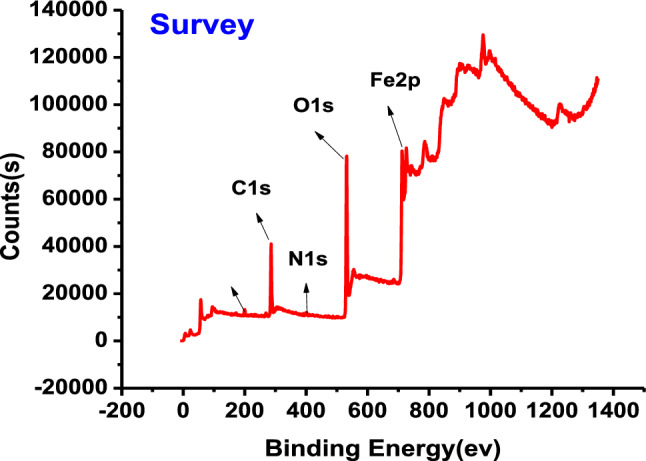
The XPS survey spectrum results of Furosemide compound adsorbed on the CS in 1 M HCl at 298 K.

**Figure 14:**
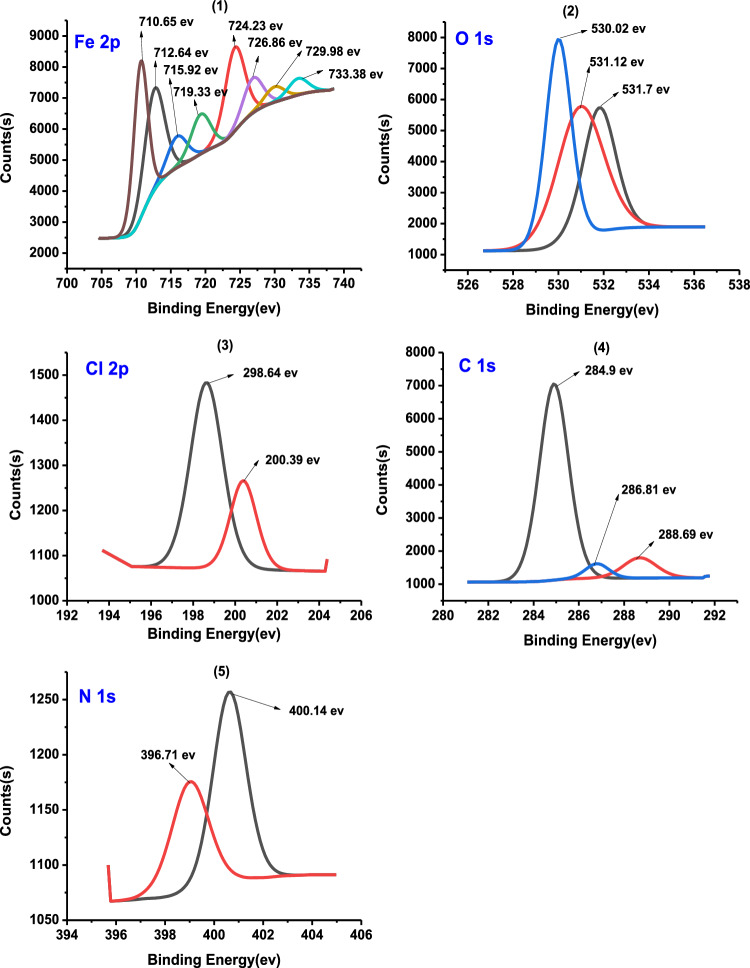
High-resolution X-ray photoelectron profiles of (1) Fe 2p, (2) O 1s, (3) Cl 2p, (**4**) C 1s and (5) N 1s for (CS) in presence of 300 ppm furosemide compound in 1 M HCl at 298 K.

It is an ideal system for predicting the adsorbed atoms on the metal surface. XPS investigation of carbon steel after immersion in 1 M HCl with 300 ppm Furosemide at 25 °C for particular atoms such as C, O, N, and Fe. The obtained results are displayed in Figs. 13 and 14. The position of the peak of this curve represents the adsorbed atoms and the intensity represents the concentration of the adsorbed element^74–77^. The C steel bar is released in a corrosive medium (1 M HCl) for 24 h in the presence of 300 ppm of the investigated compound. Table 10 summarizes the results of the data analysis.

**Table 10:** Lists the binding energies of various surveys and the expected bonds for each.

Scan type	Binding energies peaks (ev)	Peak refers to
Fe 2p	710.65	Fe 2p3/2 (Fe^+2^oxide state of FeO)
	712.64	Fe 2p3/2 (Fe^3+^ oxide state of Fe_2_O_3_ or FeCl_3_)
	715.92	Fe 2p3/2 (Fe^2+^ satellite)
	719.33	Fe 2p3/2 (Fe^3+^ satellite)
	724.23	Fe 2p1/2 (Fe^2+^ oxide state of FeO)
	726.86	Fe 2p1/2 (Fe^3+^ oxide state of Fe_2_O_3_)
	729.98	Fe 2p1/2 (Fe^2+^ satellite)
	733.38	Fe 2p1/2 (Fe^3+^ satellite)
O 1s	530.02	Fe_2_O_3_
	531.12	C–O
	531.7	C=O or C–O–C
Cl 2p	198.64	Cl 2p3/2
	200.39	Cl 2p1/2
C 1s	284.4	C–C
	285.9	C–(O, N) or C–Cl bond in inhibitor
	288.6	O–C=O
N 1s	396.71	(Fe–N–C) and (–C=N)
	400.14	(–N–H)

#### FT-IR spectroscopy

**Figure 15:**
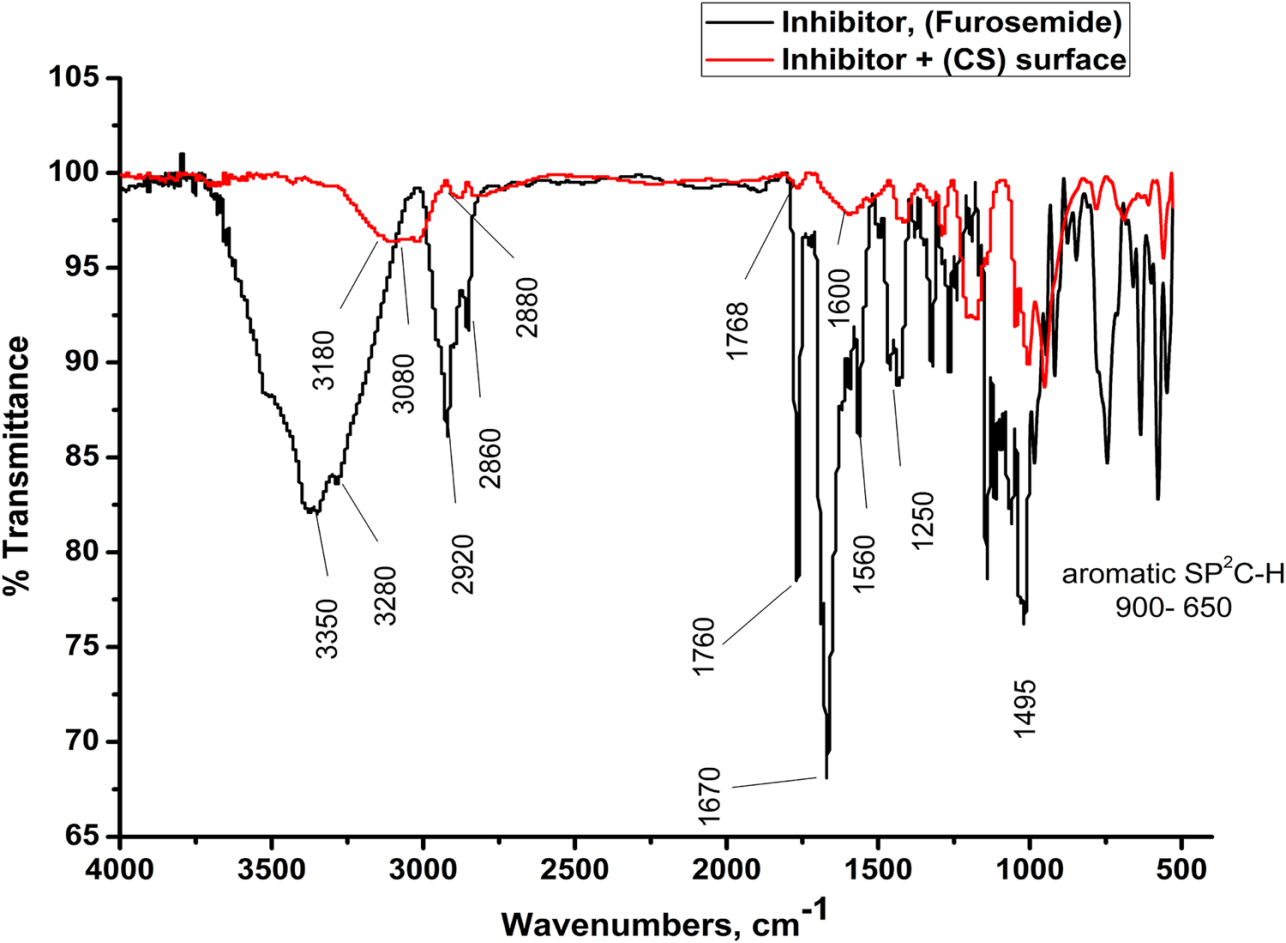
FT-IR spectra of pure Furosemide and of CS metal surface in1.0 M HCl with 300 ppm of Furosemide compound for 6 h at 298 K.

FT-IR spectra, as shown in Fig. 15, illustrate the functional groups of the solutions and their behaviour on the metal surface following adsorption with amazing precision. Table 11 shows how the FTIR results could be interpreted. Figure 15 depicts the FT-IR spectra of pure inhibitor and layers formed on CS samples after dipping in 1.0 M HCl for a day in the presence of 300 ppm Furosemide. When the spectra of the inhibitor solution and the spectra of the CS surface after immersion are compared, the two spectra have identical features, suggesting that the compounds were adsorbed on the CS^78^. The results demonstrate the interference mechanism between the inhibitor and the carbon steel surface. The shifting and removal of peaks in the spectrum after immersion demonstrated that the inhibitor interacted with the carbon steel surface via functional groups.

**Table 11:** FTIR spectra of Furosemide pure solutions and the spectra of the CS surface after inhibitor adsorption CS metal surface in1.0 M HCl with (300 ppm) of Furosemide compound for 6 h at 298 K.

Comp	Solution peaks and frequencies (cm^−1^)	Frequencies refer to	Shifting, missing and new peaks and frequencies (cm^−1^) after adsorption
Furosemide	3350, 3280	Stretching primary amine –NH_2_	Missed
	–	Stretching alcoholic for solvent O–H	3200
	–	Stretching secondary amine –NH_2_	3130
	2920	Carboxylic acidic stretching O–H	2882
	2850	Stretching SP3 –C–H	2815
	1766	Stretching carboxylic acidic stretching O–H group (–C=O)	1768
	1688	Stretching (C=C)	Missed
	1560	Bending –N–H–	1597
	1150	Alkoxy C–O	1150
	900–650	Aromatic SP2 C–H bending	950–550
	790	Ortho di-substituted	785
	780	Para di-substituted	770
	659	Meta di-substituted	691

### Quantum chemistry and statistics parameters

**Figure 16:**
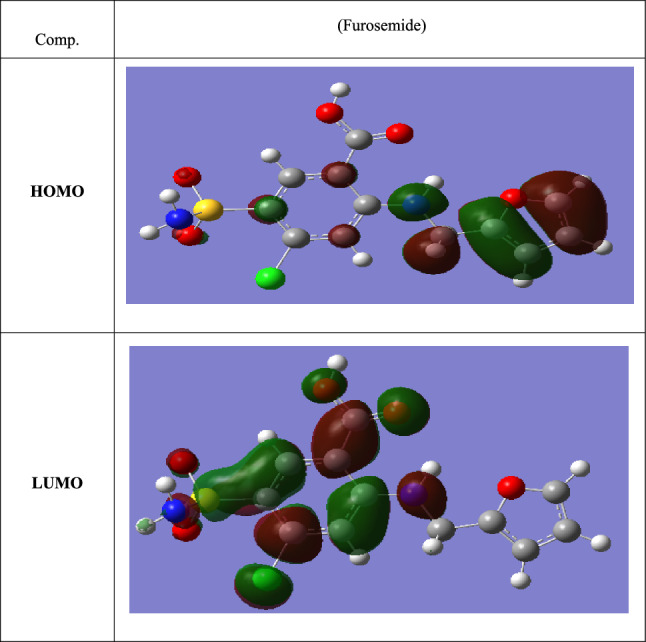
HOMO and LUMO electronic densities distributions of Furosemide compound.

Figure 16 represents the HOMO and LUMO electronic densities distributions of the Furosemide compound. According to the literature, the inhibitor molecule's E_HOMO_ is a direct indication of its tendency to donate electrons to acceptor atoms, whereas E_LUMO_ is a measure of taking electrons into its LUMO from a compatible donor molecule. ΔE_gap_ indicates the difference between E_LUMO_ and E_HOMO_. It is widely known that low values of ΔE_gap_ afford good inhibitory performances, since the energy for removing an electron from the final occupied orbital is low^79^. The low value of ΔE_gap_ for the investigated inhibitor reveals the strong inhibition efficacy of the examined compound, which indicates good adsorption of the explored inhibitor on the mild steel surface^79^. Equation (14) is used to estimate the fraction of electrons transported (ΔN) from an inhibitor molecule to the metallic surface:

14 $$ \Delta {\text{N}} = (\chi_{{{\text{Fe}}}} - \chi_{{{\text{inh}}}} )/{2}(\eta_{{{\text{Fe}}}} + \eta_{{{\text{inh}}}} ) $$

whereas χ_Fe_ and χ_inh_ represent the overall electronegativity of the iron atom and the inhibitor molecule, respectively, η_Fe_ and η_inh_ imply the absolute hardness of the iron atom and the inhibitor molecule. χ_Fe_ is 7 eV mol^−1^ and η_Fe_ iron is 0 eV mol^−1^.

These quantities are directly associated with the electron affinity (A) and ionization potential (I) of the relevant chemical systems shown below. Electronegativity (χ) and hardness can be calculated from Eqs. (15) and (16), respectively

15 $$\chi = (I+A)/2$$

and

16 $$\eta = (I-A)/2$$

Using Koopman's theorem, I = − E_HOMO_ and A = − E_LUMO_. It has been found that a value of ΔN smaller than 3.6 indicates higher inhibitor efficacy due to the inhibitor's electron-donating capacity to the iron surface^80^. The computed ΔN value of 1.437 supports the experimental interpretations. Figure 16 depicts a schematic illustration of Furosemide inhibition.

Table 12 provides quantum chemical characteristics that predict the relationship between the molecular construction of inhibitor particles and their effect against CS corrosion, in addition to the major effect on electronic interference between CS and inhibitor solution.

**Table 12:** Quantum chemical parameters for Furosemide compound in liquid phase.

Parameter	*Compound* *(*Furosemide)
*E*_*HOMO*_ (eV)	*−* *6.11*
*E*_*LUMO*_ (eV)	*−* *2.03*
*∆E*_*gap*_ (eV)	*4.08*
*µ(debye)*	*6.18*
*I* = *−* *E*_*HOMO*_	*6.11*
*A* = *−* *E*_*LUMO*_	*2.03*
χ = (I + A)/2	*4.07*
η = I − A/2	*2.04*
*σ* = *1/η*	*0.49*
*ΔN*	*1.437*

One of the most vital features, which is related to the polarity of the inhibitive particle, is the dipole moment^81^. Table 12 displays that the calculated value of the dipole moment (*μ*_inh_ = 6.18 Debye) for the studied molecule is higher than that of H_2_O (*μ*H_2_O = 1.88 Debye). The number of inhibitory sites on the metal surface in the corrosive environment is increased by this high dipole moment value. As a result, the strong dipole–dipole interactions between the inhibitor and the metal surface, as demonstrated in Table 12 and Fig. 16, are responsible for the inhibitor's high dipole monent. Certain parameters, such as global hardness (η) and softness (σ), are linked to the molecule's selectivity and reactivity. As per the Lewis theory of acid/base and Pearson's hard/soft acids and bases^82^, more reactive and greater ∆E values are associated with hard molecules. Based on Table 12, it can be inferred that Furosemide has a greater σ value, indicating a softer nature. Furosemide would therefore be more likely to provide electrons to CS.

## Molecular electrostatic potential (MEP)

**Figure 17:**
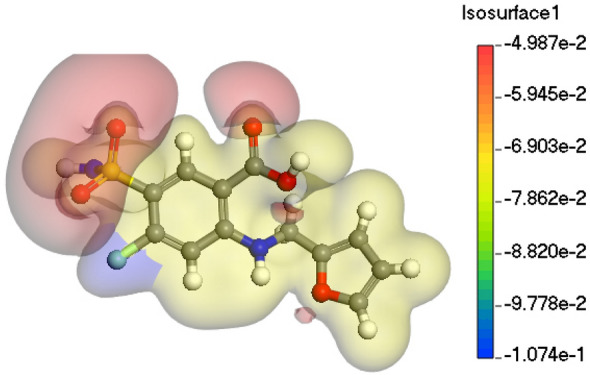
MEP surface of Furosemide compound.

The molecular electrostatic potential (MEP) is used to determine a species' relative reactivity under nucleophilic and electrophilic attack. DFT calculations were used to analyze the compound's MEP surface, using the optimized structure and B3LYP/6-31Gþ(d,p) basis set. MEP is used to examine the relationship between a compound's structure and its physicochemical properties, as well as interactions with metal surfaces. MEP modeling was recognized as a highly effective method^83^. Figure 17 depicts the electrostatic potential surface mapped for Furosemide. The MEP chart shows discrete zones with negative and positive potential. The MEP chart depicts how potential varies with colour is transite from blue to green, yellow, orange, and red^84^. The polarization effect is plainly seen in the compound. The negative potential regions of the MEP are confined over the electronegative atoms (oxygen, nitrogen, and sulfur). The green–blue regions represent positive potentials, indicating they receive electrons from the metal surface. Conversely, the red-yellow zones show negative potentials associated with electrophilic and nucleophilic performance.

## Mechanism of inhibition

**Figure 18:**
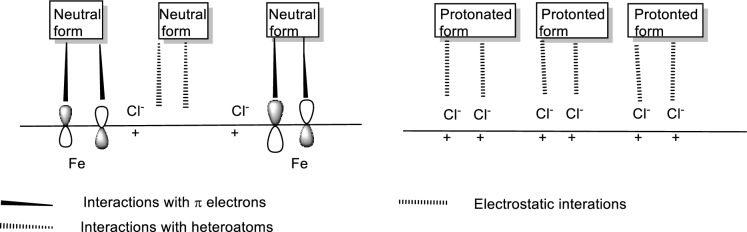
Representation of the different modes of adsorption of Furosemide drug on the CS surface.

The efficacy of the investigated compound as an inhibitor for CS corrosion in 1 M HCl is examined. Concentration, the number of active sites and their charge densities, molecule mass, and environmental stability are factors that influence the inhibition process^85–88^. In a 1 M HCl solution, the examined compound could exist as neutral molecules or in protonated form (cations). The compound may adsorb on the CS/HCl interface via one or more of the following mechanisms: (1) electrostatic interaction of the protonated form with adsorbed chloride ions; (2) donor–acceptor interactions between the electrons of the aromatic ring and vacant d orbitals of surface iron atoms; and (3) interaction between unshared electron pairs of inhibitor heteroatoms and vacant d orbitals of iron surface atoms. In one approach, the neutral inhibitor may be adsorbed on the CS surface via the chemisorption mechanism^89^. The inhibitor molecules can also adsorb on the surface via donor–acceptor interactions between the p-electrons of the heterocyclic ring and the surface iron's unoccupied d orbitals. In another way, because it is well known that the CS surface bears a positive charge in acid solution, the protonated inhibitor finds it difficult to approach the positively charged CS surface due to electrostatic repulsion. Because the CS surface is positively charged, anions (Cl^−^ ions) in an aqueous hydrochloric acid solution are adsorbed on it. The CS surface becomes negatively charged after Cl^−^ ion adsorption. As a result of electrostatic interactions with the Cl^−^ ions already adsorbed on the surface of CS, the protonated positively charged form of inhibitor is adsorbed on the surface of CS. As a result, adsorbed Cl^−^ ions and protonated inhibitor work together. Figure 18 shows a graphic representation of the adsorption of the investigated inhibitor molecule on the CS surface.

## Conclusions

1. Furosemide is safe for people; it can be used as a green corrosion inhibitor to protect CS from corrosion in 1 M HCl.
2. As Furosemide medication concentrations are increased, the efficiency of corrosion inhibition improves, reaching 90.5% at 300 ppm and 318 K.
3. The potentiodynamic polarisation investigations revealed that E_corr_ values are slightly affected, indicating that the Furosemide drug inhibitor is a mixed-type inhibitor.
4. The adsorption of Furosemide medication on the Cs surface, as well as the formation of a barrier film that isolates the metal from corrosive environments prevent corrosion.
5. The inhibitor's adsorption followed the Temkin adsorption isotherm.
6. According to the studies from different procedures that are in good agreement with one another, Furosemide medicine prevents CS corrosion in 1 M HCl and delays the process of iron breakdown in this environment.
7. The quantum chemical description of the inhibitory activity of Furosemide confirms the experimental data.

Comparison between the results obtained from this study and other studies on Furosemide and its related compounds are illustrated in Table 13.

**Table 13:** Comparison of our outcomes with previously reported studies on the employment of Furosemide and its derivatives as anti-corrosive agents.

Inhibitor	Optimum dose	Metal substrate	Corrosive media	Temp	Inhibition efficiency (%)	References
Furosemide	300 ppm	C-steel	1 M HCl	298 K	87.6	This study
Furosemide	300 ppm	Zinc	2 M HCl	298 K	81.32	^90^
Furosemide	14 × 10^–4^ M	Mild steel	1 M HCl	303 K	84.73	^91^
Hydrochlorothiazide	15 × 10^–5^ M	C- steel	2 M HCl	303 K	81.8	^92^
Acyl hydrazide containing sulfonamide moiety (p-TSAH)	1 × 10^−3^ M	Mild steel	1 M HCl	298 K	93.73	^93^

## Data Availability

The following instruments were used in this study. Potentiostat/galvanostat/ZRA (Gamry Reference 3000) in Faculty of Science, Port Said University. SEM, The analysis was performed using a Scanning Electron Microscope (JEOL JSM-5500, Japan). Faculty of Agriculture, Mansoura University, Egypt. AFM device model is Thermo Fisher Nicolet IS10 (scanning probe microscope) was employed at Nanotechnology Laboratory, Faculty of Engineering Mansoura University, Egypt. The XPS test was performed using K-ALPHA (Thermo Fisher Scientific, USA). Central Metallurgical Research Institute (CMRDI), Helwan, Egypt. FTIR spectra were performed using PerkinElmer 1600 spectrophotometer, Faculty of Pharmacy Mansoura University, Egypt.
